# Biogas Digestate and Sewage Sludge as Suitable Feeds for Black Soldier Fly (*Hermetia illucens*) Larvae

**DOI:** 10.3390/toxics12060414

**Published:** 2024-06-05

**Authors:** Jana Kofroňová, Abir Melliti, Radek Vurm

**Affiliations:** Department of Environmental Chemistry, Faculty of Environmental Technology, University of Chemistry and Technology Prague, Technická 5, 166 28 Prague, Czech Republic; mellitia@vscht.cz (A.M.); vurmr@vscht.cz (R.V.)

**Keywords:** insect, black soldier fly, waste, heavy metal, animal feed

## Abstract

*Hermetia illucens* larvae can use organic wastes as a substrate, which makes them an interesting potential feed. However, waste may contain heavy metals, which are limited in feed. Here, we investigated the ability of *H. illucens* to grow on organic wastes and measured their heavy metal bioaccumulation. The larvae were fed with food waste, biogas digestates, and sewage sludge. When the first adult fly was visible, the tests were stopped and the larvae immediately processed. The samples (wastes before use, larvae after feeding) were analysed for mineral nutrient and heavy metal content using AAS and ICP-OES, respectively. The results show that the weight of the larvae fed with food waste increased sevenfold, which was broadly in line with expectations. Those fed with sewage sludge and digestate from biogas station increased threefold. While the larvae fed with sewage sludge exceeded the limits for heavy metals, particularly Cd and Pb, in feedstock, those fed with biogas digestate and food waste did not. These findings add to the literature showing the suitability of different wastes as *H. illucens* feed, and the importance of excluding waste contaminated with heavy metals from larvae intended for use as animal feed, or else diverting these larvae to non-feed uses.

## 1. Introduction

As the global population and living standards rise, the organic waste generated in relation to food consumption is gaining more attention [1,2]. Although organic wastes, such as biogas digestate and sewage sludge, can serve as fertilisers or composts in agriculture, their composition and the amount produced are serious issues [3,4,5]. Both types of waste are usually sent to the landfill or for energy recovery, which are the least preferred options in the waste management hierarchy [6,7,8].

In the last decades, focus has been placed on the use of black soldier flies (BSF), *Hermetia illucens* (Diptera: Stratiomyidae), in organic waste processing. The production of these larvae is relatively simple, and they have been mainly used in waste management in developing countries [9,10]. They are capable of consuming a wide variety of organic wastes, even human faeces [11]. Studies into the use of BSF larvae for organic waste processing have mainly focused on food waste as their feed; analyses of various parameters, such as lipid, amino acid, vitamin and mineral content, have confirmed its suitability [12,13,14,15,16,17]. However, the number of studies that have investigated other types of wastes, such as agricultural and special waste, is significantly lower.

BSF larvae are not only able to decompose organic substances, but also various toxins produced by bacteria or fungi [16,18]. Under specific conditions (temperature, moisture, etc.), the larvae fed with wastes can develop to the prepupae stage from days 14 to 40 [19,20]. The prepupae live on fat reserves, becoming adult flies after a further two weeks [21]. Furthermore, the larvae themselves can be used as feed; in which case, their life cycle should be terminated just before the prepupae stage as this is when nutrient composition is at its highest [22].

Although larvae have natural immunity, their accumulation of toxins and toxicants can cause problems if they are to be used as feed for livestock [23,24,25]. Under European Union (EU) feed legislation, rearing larvae on materials of animal origin is prohibited, with a few exceptions such as milk, eggs, honey, and others. The EU has prescribed legal limits for various pollutants, such as heavy metals and aflatoxins in ‘Council directive 2002/32/EC’ [26]. When manure was used as a feed for larvae, analyses revealed the accumulation of heavy metals, especially As, Cd, Hg and Pb, but the larval growth rate was not significantly affected [27,28]. Even larvae reared on polluted corn leaves assimilated Cd and Zn [29].

In terms of waste origin, which must be taken into account, the risk of substance accumulation is more significant when biogas digestates and, particularly, sewage sludge is used [5,30]. Sewage sludge from wastewater treatment plants usually contains not only organic matter and a high nutrient content but also chlorine compounds, metals, pharmaceuticals, and pathogenic organisms [31,32,33]. By comparison, digestates, while not being as rich in organic matter, may have lower amounts of some toxic substances such as heavy metals or pharmaceuticals. In the case of using both organic wastes as feed for larvae in a more efficient manner, it is necessary to determine their effects on larval growth and composition, especially regarding the pollutants which may reduce their reusability.

In this paper, we focus on the suitability of food waste, biogas digestate, and sewage sludge as a feed for *H. illucens* larvae in terms of the larval growth, larval development, and mineral composition of larvae and feed. With regards to legislative limits, the heavy metal content of the larvae was analysed.

## 2. Materials and Methods

### 2.1. Substrates

Tests were carried out with three types of organic waste, namely food waste (FW), two biogas digestates (BG1 and BG2), and a sample of sewage sludge from a wastewater treatment plant (S). All samples originated from the Czech Republic. Food waste was collected from a nearby school canteen and processed into the final substrate using an electric kitchen composter (GG-10, Oklin, Bangkok, Thailand) which reduced the volume of waste by about 90%. Food waste was manually sorted for potential plastic contamination prior to shredding. Biogas digestates were collected from two different biogas plants that use mostly agricultural waste such as maize silage, grass haylage, and cattle slurry in wet fermentation processes to produce biogas. Sewage sludge was collected from a wastewater treatment plant that treats municipal and industrial wastewater using mechanical and biological processes. Distilled water was added to ensure suitable moisture in the food waste. The sludge and digestate samples utilised were excessively aqueous, requiring pretreatment before further use. Consequently, a small quantity of coconut substrate (15% *w*/*w*) was added into the digestate samples. While this is not digestible by the larvae, it does provide a more appropriate level of viscosity, which should ideally lie between 70 and 80% [34], but even higher levels of moisture do not have a significant impact on the survival rate of larvae [35]. For the sludge experiments, coconut substrate was not employed; rather, centrifuged sludge was used.

### 2.2. Experimental Design

Black soldier fly larvae were obtained from a private breeder. Prior to testing, the larvae were washed with distilled water and left in a box for 24 h to empty their gastrointestinal tract. Seven-day-old larvae (approx. 700 per replicate) were weighed into the plastic containers (80 × 90 × 160 mm) which were adjusted with the metal grid on the lid to prevent escape after the previous addition of waste sample (100 mg of dry weight/larva). The containers were placed in an incubator with a temperature set at 28 °C and a 14:10 light cycle. The larvae were fed regularly, every two or three days, and demineralised water was added according to the rate of evaporation to maintain at least 70% humidity. Experiments were carried out in triplicates.

Feeding of larvae was stopped when the first adult flies were observed, and the development time varied between samples. Larvae were picked by hand with tweezers, rinsed several times with demineralised water, and dried with paper towels. After drying, the larvae were weighed and placed in an ultra-low freezer at −80 °C prior to lyophilisation. After 24 h lyophilisation (Alpha 2-4 LD plus, Martin Christ, Osterode am Harz, Germany), larvae were crushed with a pestle in a mortar and used for subsequent analyses.

### 2.3. Mineralisation and Elemental Analysis

Larvae and substrates were analysed for mineral nutrient content and heavy metal content after lyophilisation. Powder samples were placed in beakers with a mixture of hydrochloric acid (37%) and nitric acid (65%) in ratio of 3:1 and heated on a heating plate at 80 °C until complete mineralisation. After filtration and dilution, the mineralised samples were analysed by atomic absorption spectrometry (SensAA, GBC Scientific Equipment, Keysborough, Australia) for sodium (Na), potassium (K), calcium (Ca), and magnesium (Mg), and by ICP-OES spectrometry (iCAP 7400, Thermo Scientific, Waltham, MA, USA) for total phosphorus (P), manganese (Mn), iron (Fe), copper (Cu), zinc (Zn), aluminium (Al), and heavy metals such as lead (Pb), cadmium (Cd), chromium (Cr), and nickel (Ni). The nutrient contents were expressed as the amount of compound per kg of dry matter.

### 2.4. Data Analysis

The experiments and sample analyses were performed in three independent biological replications. The element content data are presented as mean ± SD (n = 3). One-way analysis of variance (ANOVA) was performed to test the differences among larval treatments and Student’s t-test was used post hoc to determine the significance of the differences at *p* < 0.05, followed by Bonferroni correction for multiple comparison.

## 3. Results

### 3.1. Composition of Substrates

Food waste had the highest amounts of calcium and sodium among the macronutrients studied, more than double the levels found in digestates and sludge (Table 1). Its calcium to phosphorus ratio was over 11, whereas the ratios for the other substrates were significantly lower at around 0.8 for sludge, which also showed the highest phosphorus value, and between 1.3 and 1.6 for both digestates. The digestates had relatively high potassium contents, with more than tenfold the amount found in sludge. The quantities of micronutrients present in food waste were notably lower than those discovered in digestates and sludge, even by a factor of thousands. The greatest levels of micronutrients were discovered in sludge, where notably higher Fe, Cu, and Zn quantities were observed in comparison to digestate. Conversely, the quantity of Mn present in sludge was lower than in digestates and almost double compared to BG1. The quantities of micronutrients in the digestates were mostly equal, except for the Fe content which exhibited a threefold difference. The substantial quantities of heavy metals followed an analogous trend to that of the micronutrients. The maximum amounts were observed in the sludge, notably for Ni and Cr. A few elements, particularly As, in the food waste and coconut were below detection limits. Heavy metal content in the digestates was mostly around 2 mg/kg.

### 3.2. Larval Growth Performance

The initial dry weight of the larvae matched that of the ones raised on food waste (Table 2). Conversely, although the moisture content was maintained with minimal fluctuations, larvae fed with digestates and sludge exhibited only half the dry weight. Larval development rates were comparable between the food waste and digestates and took about a month or less. 

A difference of one week in development at the final stage was observed between the two digestates. Nearly twice the duration of growth was required in BG2 digestate, the larvae in the sludge attained an equivalent weight of around 47 mg per individual larvae. Results demonstrate that fewer larvae in ten grams resulted in higher individual weight and better development. Larvae reared on food waste experienced a sevenfold increase in weight compared to their starting point, while the larvae reared on BG2 digestate and sludge increased their weight by over threefold.

### 3.3. Mineral Composition of Hermetia illucens

Table 3 displays the mineral composition results of *H. illucens* after testing with different substrates. The findings indicate that larvae raised on food waste had the lowest mineral content per kg dry weight, even though some minerals, such as Ca or Na, had the highest content in the analysis of the original substrates (Table 1). The mineral content of larvae from both digestates was quite similar in magnitude, despite a seven-day difference in larval development (Table 2), indicating an anticipated increase. Although larvae reared on digestate BG1 had slightly greater Ca and P content than those on BG2, they had lower K and higher Mg content. This indicates a distinct contrast from the substrates, where BG1 had higher values for all minerals compared to BG2. Considerable accumulation of Ca, Mg, and all trace minerals was observed in digestate-fed larvae in contrast to larvae fed with food waste, which had lower values for all minerals. Larvae from digestates also showed more than fivefold higher levels of Mn, Fe, and Cu compared to larvae reared on food waste. When analysing the digestate-fed larvae and sludge-fed larvae, only Ca showed a significant difference in terms of macromineral content, with a twofold increase. Other macrominerals have similar content, and even the amount of Mg is lower than that of BG1. When studying micromineral content in sludge-fed larvae, commensurate trends as those in the substrate itself were observed. Compared to larvae from the digestate, higher amounts were found for most elements, with up to a tenfold increase for Cu and an eightfold increase for Fe. The substrates also had a considerable impact on the Ca/P ratio, which was lowest for larvae from food waste and highest for those from sludge.

### 3.4. Heavy Metal Content in Hermetia illucens

The heavy metals that were analysed and pose health hazards with toxic effects comprised cadmium (Cd), lead (Pb), chromium (Cr), nickel (Ni), and arsenic (As). Due to detection limitations, the amount of As in larvae from food waste and digestates, and the amount of Ni in BG2 larvae could not be measured. Larvae fed with sludge recorded the highest levels of all heavy metals analysed (Table 4), which is consistent with the results of the original substrate analysis. Food waste contained the lowest levels for almost all metals analysed. The digestate-fed larvae had very similar values of around 1 mg/kg for all heavy metals. The amounts of Cd were below 0.5 mg/kg. However, the analysis of the larvae reared on BG2 digestate showed higher levels, except for Ni, and both concentrations exceeded the amount of 1 mg/kg for Pb.

## 4. Discussion

This study examined the capability of larvae to consume diverse forms of organic waste, namely food waste, biogas digestate, and sewage sludge. The mineral composition and heavy metal contamination were compared across these wastes. The results confirmed that biogas digestate and sewage sludge can be viable substrates for *H. illucens*, but only if the waste origin and nature are taken into account. Larvae aged seven days were utilised for the tests, which is deemed more efficient when fed with less digestible waste [36]. The larvae’s preference for various material structures differs during the developmental stages. While a reduction in feed does not lead to significant mortality in the larvae, it does result in an extended development period [37]. This is due to the slower establishment of the minimum energy reserves required for pupal transition. Additionally, it reduces the dry matter yield at maturity [38]. Suitable conditions must be maintained to guarantee a consistent rearing process that transforms waste into biomass [39]. The larval rearing substrate has a significant impact on the mineral composition of the resulting larvae. Certain parameters (e.g., P, Mg, or K content) are more affected by this influence than others (e.g., Na) [40,41]. However, several studies indicate that the capability to thrive and increase in biomass on diverse substrates is not limited to food waste and animal feed [41,42,43], but also extends to animal manure [44,45,46] and faeces [23,38,47].

### 4.1. Larval Growth Performance

The larvae reared on food waste showed the highest weight gain and quicker development within one month, possibly owing to its good diet composition with little variance in nutritional content. Typically, food waste holds a broad range of edible materials such as leftover cereals, meat, fruits, and vegetable residues [13,48]. Hence, it comprises proteins, fats, fibres, and minerals in varying proportions [12,45]. It should also not contain more contaminants than, for example, sludge [49,50], which could affect larval development. Heavy-metal-containing substrates lead to growth disturbances in larvae, resulting in lower weight and feed conversion ratio [18]. In addition to heavy metals, the substrate commonly contains contaminants such as microorganisms and pesticides. Although insect larvae possess antimicrobial properties against certain human pathogens [51,52], they also contain microorganisms and chemical contaminants that can be transferred to the product during subsequent use [23,53,54]. The larvae fed with digestate and sludge did not increase in biomass as much as those fed with food waste. However, their biomass did increase threefold compared to beginning and in one case, the development time of the larvae fed on digestate was even shorter. Pas et al. [55] has noted that digestate has lower nutritional value in terms of protein and fat than food waste. However, its mineral content is significantly higher. The higher utilisable nutrient content in digestate leads to higher larval yields [56]. Improving the digestibility and utilisation of digestate can be accomplished through degradation, particularly by minimising the impact of cellulose [55].

### 4.2. Mineral Composition of Hermetia illucens

Macrominerals, including calcium, phosphorus, magnesium, potassium, and sodium, are present in complete feed at a rate of grams per kilogram of dry matter, playing a crucial role in livestock’s proper growth and enhancing their overall quality. In adequate proportions and ratios, they participate in numerous physiological processes in animals such as energy metabolism, bone maturation, cellular structures, and significantly contribute to the eggshell growth and durability in laying hens [57,58,59]. The Ca/P ratio is a crucial factor for feed. It is recommended to maintain a ratio of 10:1 to 15:1 for hens producing table eggs [60] and between 1.5 and 1.8:1 for channel catfish [61]. The Ca/P ratios we observed in the larvae that were fed with food waste and the digestates were almost within the specified ranges, with 1.54 for food waste and 3.82, 4.02 for digestates. But rearing requirements depend on various factors, including season, developmental period, and rearing system [62]. At the same time, excessive elemental content such as Ca may elevate stomach pH and make one more prone to infection [63].

Larvae fed with both digestates and the sludge exhibited considerably higher levels of different minerals, particularly Ca, Na, and P, when compared to those fed with food waste. The elements within the substrates adhere differently and thus have varying accessibility to the larvae. Calcium was present in noticeably higher concentrations compared to other minerals in almost all larvae tested, which is beneficial for their subsequent utilisation. The sole exception was observed in food waste where the concentration of K exceeded that of Ca. The elevated levels of Ca may be attributed to the presence of its higher levels in BSF larvae from the beginning of their life since it is integrated as calcium carbonate in their exoskeleton [64]. Furthermore, its quantity increases in the prepupal and pupal stages. The recorded Ca levels differ in the published literature [12,15,40]. When compared to pure fruit and vegetable waste, our detected levels in food waste were over twofold lower [12]. However, when compared to restaurant and kitchen waste, our levels were over threefold higher [15,40]. The amount of phosphorus in restaurant and kitchen wastes in these studies did not differ significantly from our observed value of around 4 g/kg. The analyses of larvae fed with the sludge demonstrated comparable macromineral values to those in the digestates, with the exception of Ca, which had nearly double the concentration for sludge. Conversely, the Ca values were almost threefold lower in the digestate-fed larvae than in a similar study conducted by [40], while the levels of Mg, Na, and P were twice as high. The elevated Ca concentration of digestates could be attributed to the initial waste utilised for anaerobic digestion, which could have comprised dairy products [55].

The analyses of copper, manganese, zinc, and iron were conducted among the trace minerals. The results suggested that Zn content increased in substrates with higher concentration in the beginning such as sludge, where more than 3 g/kg resulted in almost 1 g/kg in larval mass. Extraneous Zn supplies resulted in an accumulation in the final developmental stage, without any observed toxic effects at levels of approximately 420 mg/kg [29]. Zinc’s significance in insects as a constituent of enzymes and DNA-binding proteins has also been established [65]. However, the zinc levels detected in the larvae were high and may have caused adverse effects, leading to reduced yields compared to food waste. Nevertheless, insects have a similar function to other organisms in that they accumulate or excrete minerals at higher concentrations. The Cu, Zn, and Fe content in larvae fed with the digestates were several times lower than those in the sludge, while Mn content was over twice as high. Spranghers [40] found that prepupae reared on digestate had lower levels of certain elements. The amount of Cu was less than twofold (10 mg/kg), while Zn was more than fivefold (50 mg/kg). Similarly, Mn and Fe values were comparable, measuring at 380 and 430 mg/kg, respectively. It should be noted that just because one mineral accumulates well, it does not necessarily mean that the entire mineral composition is suitable [12].

### 4.3. Heavy Metal Content in Hermetia illucens

Heavy metals are frequent contaminants in the feed industry, and they are subject to limits. According to Directive 2002/32/EC of the European Parliament and the Council regarding undesirable substances in animal feed, the authorised thresholds in finished feed are set at 2 mg/kg for arsenic, 5 mg/kg for lead, and 0.5 mg/kg for cadmium [26]. The concentration of heavy metals in the larvae is contingent on the substrate. When sludge was used as a feed, the high initial concentration in the substrate (213.8 mg/kg of lead and 10.7 mg/kg of cadmium) caused a significant accumulation of cadmium (7.3 mg/kg) and lead (54 mg/kg). This accumulation greatly exceeded the applicable limits and rendered sludge an unsuitable substrate for use as feed. By contrast, although the digestate contained a higher concentration of heavy metals than the food waste, the accumulation in the larvae did not exceed the limits, making it a potentially suitable feed for larvae for future use. Our findings align with similar studies for various food waste [54,66,67,68,69,70], where the levels of Pb in larvae for food waste range from 0.08 to 1.5 mg/kg, while Cd ranges from 0.02 to 7.9 mg/kg, and Cr ranges from 0.08 to 3 mg/kg. Previous studies have shown that Cd and Pb can accumulate in larvae [18,28]. Although higher levels of Cd were observed, no significant difference in development was found [28,29,71]. Nonetheless, the presence of heavy metals may lead to a slight increase in oxidative stress and potentially impact the health of the microbiome [71,72]. At higher concentrations of Cr, there is a greater accumulation in the larval stage than in the pupal stage [73]. This could be attributed to the cessation of feeding at this stage [20]. A similar pattern was also noted for Cd and may suggest an enhancement in the defence mechanism against heavy metals as development progresses [29].

While some previous studies have examined comparable substrates to ours, noticeable disparities in composition were observed in the final outcomes for specific minerals and heavy metals. Potential variables that might have influenced these disparities include substrate composition, substrate supply rate, larval origin, larval age, instar analysis at different stages, experimental design, and rearing conditions [18,28]. Black soldier fly larvae are increasingly demonstrating their potential as a viable feed ingredient across various animal species. Additionally, enhancing the quality and availability of nutritional values through the inclusion of, for example, vitamin A supplementation and the manipulation of the larval diet are worth investigating further [38,74,75].

## 5. Conclusions

We have shown that *H. illucens* larvae can be successfully reared on typically landfilled organic wastes. Interesting results were observed not only for food waste but also for the biogas digestates and sewage sludge, which are considered as problematic for subsequent processing. All the tested organic wastes increased larval growth, which may be attractive for their effective utilisation in terms of waste management hierarchy. In the case of biogas digestate, the concentration of heavy metals in final larval stages were below the legal limits, enabling their possible use as an alternative feed for livestock, after fulfilment of all feed requirements. For sewage sludge, which is more contaminated, the higher accumulation of heavy metals (limits exceeded) did not show a significant decrease in analysed parameters, but for other pollutants or higher amounts, the results might be different. Although biogas digestate and sewage sludge have their own limitations, *H. illucens* might have potential for future use in waste prevention, biodiesel production, entomoremediation, or as a fertiliser.

## Figures and Tables

**Table 1:** Mineral and heavy metal content of different substrates used to rear BSF larvae.

Parameters	Substrate ^1^				
	FW	BG1	BG2	S	Coconut
Dry matter (%)	97.1	5.5	6.1	6.8	98.0
Macrominerals, g/kg DM					
Ca	22.83	11.17	7.77	10.65	1.89
Total P	1.93	6.91	5.92	13.50	0.32
Na	8.48	3.89	2.83	2.53	2.18
K	7.40	32.80	25.58	1.67	8.67
Mg	1.35	10.42	7.31	10.65	1.63
Microminerals, mg/kg DM					
Mn	8.91	323.53	206.80	152.62	45.18
Fe	64.63	2630.6	844.50	27,025.3	756.59
Cu	1.45	36.70	33.40	1509.9	4.34
Zn	28.08	309.84	235.80	3234.1	17.42
Al	23.28	354.67	243.40	1101.8	597.87
Heavy metals, mg/kg DM					
Pb	0.71	1.38	1.80	213.8	<LOQ
Cd	0.09	0.06	0.10	10.71	<LOQ
Ni	0.16	2.76	2.30	193.02	0.40
Cr	1.99	3.07	2.20	818.78	<LOQ
As	<LOQ	0.77	0.70	6.53	<LOQ

^1^ DM = dry matter; FW = food waste; BG = biogas digestate; S = sewage sludge; LOQ = limit of quantification.

**Table 2:** Initial larval and final developmental characteristics of BSF reared on different substrates.

Parameters	Start	Substrate ^1^			
		FW	BG1	BG2	S
Dry matter (%)	30.8	33.9	17.7	16.8	17.4
Larval development (days)		28	31	24	46
Number of larvae in 10 g of larval mass	700	100	265	205	215
Individual larval weight (mg)	14.3	101.0	37.8	48.8	46.5

^1^ FW = food waste; BG = biogas digestate; S = sewage sludge.

**Table 3:** Mineral composition of BSF after rearing on different substrates (means ± SD; n = 3). Statistically significant differences are marked with * (*p* < 0.05, Student’s t-test).

Parameters	BSF on Substrate ^1^			
	FW	BG1	BG2	S
Macrominerals, g/kg DM				
Ca	6.99 ± 0.61 *	29.80 ± 1.57	27.71 ± 3.18	58.67 ± 2.47 *
Ca/P ratio	1.54	3.82	4.02	6.54
Total P	4.54 ± 0.10 *	7.80 ± 0.20	6.90 ± 0.87	8.97 ± 0.47
Na	0.87 ± 0.10 *	1.97 ± 0.15	1.80 ± 0.33	2.43 ± 0.40
K	9.13 ± 0.30	10.77 ± 0.47	15.51 ± 1.50	13.17 ± 1.30
Mg	2.75 ± 0.26 *	10.63 ± 0.25	6.34 ± 0.84	9.27 ± 1.35
Microminerals, mg/kg DM				
Mn	43.67 ± 1.01 *	388.70 ± 32.36	353.27 ± 84.83	145.97 ± 13.54 *
Fe	68.33 ± 3.26 *	440.68 ± 19.07	349.57 ± 154.68	3072.6 ± 132.6 *
Cu	3.23 ± 0.21	20.95 ± 0.12	18.50 ± 2.78	212.57 ± 47.20 *
Zn	54.27 ± 5.65 *	274.65 ± 7.87	219.33 ± 24.35	976.27 ± 73.00 *
Al	<LOQ *	209.40 ± 10.97	173.73 ± 40.05	761.27 ± 86.59 *

^1^ DM = dry matter; FW = food waste; BG = biogas digestate; S = sewage sludge; LOQ = limit of quantification.

**Table 4:** Heavy metal content of BSF reared on different substrates (means ± SD; n = 3). Statistically significant differences are marked with * (*p* < 0.05, Student’s t-test).

Heavy Metals, mg/kg DM	BSF on Substrate ^1^			
	FW	BG1	BG2	S
Pb	0.43 ± 0.38	1.05 ± 0.27	1.27 ± 0.15	54.00 ± 5.41 *
Cd	0.20 ± 0.00	0.37 ± 0.06	0.40 ± 0.10	7.27 ± 1.61 *
Ni	0.13 ± 0.15	0.30 ± 0.09	<LOQ	21.07 ± 3.55 *
Cr	2.13 ± 0.45	1.26 ± 0.04	1.70 ± 0.35	146.83 ± 35.50 *
As	<LOQ	<LOQ	<LOQ	1.07 ± 0.15 *

^1^ DM = dry matter; FW = food waste; BG = biogas digestate; S = sewage sludge; LOQ = limit of quantification.

## Data Availability

Data will be made available upon request.

## References

[1] FAO (2019). The State of Food and Agriculture 2019.

[2] Gligorescu A., Toft S., Hauggaard-Nielsen H., Axelsen J.A., Nielsen S.A. (2019). Development, Growth and Metabolic Rate of *Hermetia Illucens* Larvae. J. Appl. Entomol..

[3] Iacovidou E., Vlachopoulou M., Mallapaty S., Ohandja D.G., Gronow J., Voulvoulis N. (2013). Anaerobic Digestion in Municipal Solid Waste Management: Part of an Integrated, Holistic and Sustainable Solution. Waste Manag..

[4] Mangwandi C., JiangTao L., Albadarin A.B., Allen S.J., Walker G.M. (2013). The Variability in Nutrient Composition of Anaerobic Digestate Granules Produced from High Shear Granulation. Waste Manag..

[5] Nkoa R. (2014). Agricultural Benefits and Environmental Risks of Soil Fertilization with Anaerobic Digestates: A Review. Agron. Sustain. Dev..

[6] Havukainen J., Saud A., Astrup T.F., Peltola P., Horttanainen M. (2022). Environmental Performance of Dewatered Sewage Sludge Digestate Utilization Based on Life Cycle Assessment. Waste Manag..

[7] Tyagi V.K., Lo S.-L. (2013). Sludge: A Waste or Renewable Source for Energy and Resources Recovery?. Renew. Sust. Energ. Rev..

[8] Chen T., Qiu X., Feng H., Yin J., Shen D. (2021). Solid Digestate Disposal Strategies to Reduce the Environmental Impact and Energy Consumption of Food Waste-Based Biogas Systems. Bioresour. Technol..

[9] Devic E., Maquart P.-O. (2015). *Dirhinus giffardii* (Hymenoptera: Chalcididae), Parasitoid Affecting Black Soldier Fly Production Systems in West Africa. Entomologia.

[10] Kenis M., Koné N., Chrysostome C.A.A.M., Devic E., Koko G.K.D., Clottey V.A., Nacambo S., Mensah G.A. (2014). Insects Used for Animal Feed in West Africa. Entomologia.

[11] Lalander C., Nordberg Å., Vinnerås B. (2018). A Comparison in Product-value Potential in Four Treatment Strategies for Food Waste and Faeces—Assessing Composting, Fly Larvae Composting and Anaerobic Digestion. GCB Bioenergy.

[12] Jucker C., Erba D., Leonardi M.G., Lupi D., Savoldelli S. (2017). Assessment of Vegetable and Fruit Substrates as Potential Rearing Media for *Hermetia illucens* (Diptera: Stratiomyidae) Larvae. Environ. Entomol..

[13] Kawasaki K., Hashimoto Y., Hori A., Kawasaki T., Hirayasu H., Iwase S., Hashizume A., Ido A., Miura C., Miura T. (2019). Evaluation of Black Soldier Fly (*Hermetia illucens*) Larvae and Pre-Pupae Raised on Household Organic Waste, as Potential Ingredients for Poultry Feed. Animals.

[14] Salomone R., Saija G., Mondello G., Giannetto A., Fasulo S., Savastano D. (2017). Environmental Impact of Food Waste Bioconversion by Insects: Application of Life Cycle Assessment to Process Using *Hermetia illucens*. J. Clean. Prod..

[15] Shumo M., Osuga I.M., Khamis F.M., Tanga C.M., Fiaboe K.K.M., Subramanian S., Ekesi S., van Huis A., Borgemeister C. (2019). The Nutritive Value of Black Soldier Fly Larvae Reared on Common Organic Waste Streams in Kenya. Sci. Rep..

[16] Surendra K.C., Olivier R., Tomberlin J.K., Jha R., Khanal S.K. (2016). Bioconversion of Organic Wastes into Biodiesel and Animal Feed via Insect Farming. Renew. Energy.

[17] Wang S.Y., Wu L., Li B., Zhang D. (2020). Reproductive Potential and Nutritional Composition of *Hermetia illucens* (Diptera: Stratiomyidae) Prepupae Reared on Different Organic Wastes. J. Econ. Entomol..

[18] Purschke B., Scheibelberger R., Axmann S., Adler A., Jäger H. (2017). Impact of Substrate Contamination with Mycotoxins, Heavy Metals and Pesticides on the Growth Performance and Composition of Black Soldier Fly Larvae (*Hermetia illucens*) for Use in the Feed and Food Value Chain. Food Addit. Contam. A.

[19] Booth D.C., Sheppard C. (1984). Oviposition of the Black Soldier Fly, *Hermetia illucens* (Diptera: Stratiomyidae): Eggs, Masses, Timing, and Site Characteristics. Environ. Entomol..

[20] Tomberlin J.K., Sheppard D.C. (2002). Factors Influencing Mating and Oviposition of Black Soldier Flies (Diptera: Stratiomyidae) in a Colony. J. Entomol. Sci..

[21] Abdel-Shafy H.I., Mansour M.S.M. (2018). Solid Waste Issue: Sources, Composition, Disposal, Recycling, and Valorization. Egypt. J. Pet..

[22] Veldkamp T., van Duinkerken G., van Huis A., Lakemond C.M.M., Ottevanger E.A., Bosch G., van Boekel T. (2012). Insects as a Sustainable Feed Ingredient in Pig and Poultry Diets: A Feasibility Study = Insecten Als Duurzame Diervoedergrondstof in Varkens—En Pluimveevoeders: Een Haalbaarheidsstudie.

[23] Lalander C., Diener S., Magri M.E., Zurbrügg C., Lindström A., Vinnerås B. (2013). Faecal Sludge Management with the Larvae of the Black Soldier Fly (*Hermetia illucens*)—From a Hygiene Aspect. Sci. Total Environ..

[24] Elhag O., Zhou D., Song Q., Soomro A.A., Cai M., Zheng L., Yu Z., Zhang J. (2017). Screening, Expression, Purification and Functional Characterization of Novel Antimicrobial Peptide Genes from *Hermetia illucens* (L.). PLoS ONE.

[25] Biancarosa I., Liland N.S., Biemans D., Araujo P., Bruckner C.G., Waagbø R., Torstensen B.E., Lock E., Amlund H. (2018). Uptake of Heavy Metals and Arsenic in Black Soldier Fly (*Hermetia illucens*) Larvae Grown on Seaweed-enriched Media. J. Sci. Food Agric..

[26] (2002). Directive 2002/32/EC of the European Parliament and of the Council of 7 May 2002 on Undesirable Substances in Animal Feed 2002. Off. J. L.

[27] Li Q., Zheng L., Qiu N., Cai H., Tomberlin J.K., Yu Z. (2011). Bioconversion of Dairy Manure by Black Soldier Fly (Diptera: Stratiomyidae) for Biodiesel and Sugar Production. Waste Manag..

[28] Diener S., Zurbrügg C., Tockner K. (2015). Bioaccumulation of Heavy Metals in the Black Soldier Fly, *Hermetia Illucens* and Effects on Its Life Cycle. J. Insects Food Feed.

[29] Bulak P., Polakowski C., Nowak K., Waśko A., Wiącek D., Bieganowski A. (2018). *Hermetia illucens* as a New and Promising Species for Use in Entomoremediation. Sci. Total Environ..

[30] Dean R., Suess M. (1985). The Risk to Health of Chemicals in Sewage Sludge Applied to Land. Waste Manag. Res..

[31] Sharma L.D., Sarangthem I., Thangjam R., Sadhukhan R., Oinam N., Yanglem B., Banarjee L., Anal H.J., Jatav H.S. (2022). Sewage Sludge and Its Health Risk Assessment: Opportunities and Challenges. Sustainable Management and Utilization of Sewage Sludge.

[32] Harrison E.Z., Oakes S.R., Hysell M., Hay A. (2006). Organic Chemicals in Sewage Sludges. Sci. Total Environ..

[33] Santos J.L., Martín J., Mejías C., Aparicio I., Alonso E. (2022). Pharmaceuticals and Their Metabolites in Sewage Sludge and Soils: Distribution and Environmental Risk Assessment.

[34] Cammack J., Tomberlin J. (2017). The Impact of Diet Protein and Carbohydrate on Select Life-History Traits of The Black Soldier Fly *Hermetia Illucens* (L.) (Diptera: Stratiomyidae). Insects.

[35] Khairuddin D., Ghafar S.N.A., Hassan S.N.F. (2022). Food Waste Type and Moisture Content Influence on the *Hermetia illucens* (L.), (Diptera: Stratiomyidae) Larval Development and Survival. IOP Conf. Ser. Earth Environ. Sci..

[36] Nyakeri E.M., Ayieko M.A., Amimo F.A., Salum H., Ogola H.J.O. (2019). An Optimal Feeding Strategy for Black Soldier Fly Larvae Biomass Production and Faecal Sludge Reduction. J. Insects Food Feed.

[37] Moo C.Y., Abu Hasan H. (2022). Effect of Feeding Rate on Growth Performance and Waste Reduction Efficiency of Black Soldier Fly Larvae (Diptera: Stratiomyidae). Trop. Life Sci. Res..

[38] Diener S., Zurbrügg C., Tockner K. (2009). Conversion of Organic Material by Black Soldier Fly Larvae: Establishing Optimal Feeding Rates. Waste Manag. Res..

[39] Žáková M., Borkovcová M. Comparison of Field and Lab Application of *Hermetia illucens* Larvae. Proceedings of the MendelNet.

[40] Spranghers T., Ottoboni M., Klootwijk C., Ovyn A., Deboosere S., De Meulenaer B., Michiels J., Eeckhout M., De Clercq P., De Smet S. (2017). Nutritional Composition of Black Soldier Fly (*Hermetia illucens*) Prepupae Reared on Different Organic Waste Substrates. J. Sci. Food Agric..

[41] Scala A., Cammack J.A., Salvia R., Scieuzo C., Franco A., Bufo S.A., Tomberlin J.K., Falabella P. (2020). Rearing Substrate Impacts Growth and Macronutrient Composition of *Hermetia illucens* (L.) (Diptera: Stratiomyidae) Larvae Produced at an Industrial Scale. Sci. Rep..

[42] Finke M.D. (2013). Complete Nutrient Content of Four Species of Feeder Insects. Zoo Biol..

[43] Klammsteiner T., Walter A., Bogataj T., Heussler C.D., Stres B., Steiner F.M., Schlick-Steiner B.C., Insam H. (2021). Impact of Processed Food (Canteen and Oil Wastes) on the Development of Black Soldier Fly (*Hermetia illucens*) Larvae and Their Gut Microbiome Functions. Front. Microbiol..

[44] Newton G.L., Sheppard D.C., Watson D.W., Burtle G.J., Dove C.R., Tomberlin J.K., Thelen E.E. (2005). The Black Soldier Fly, *Hermetia illucens*, as a Manure Management/Resource Recovery Tool. Proceedings of the Symposium on the State of the Science of Animal Manure and Waste Management.

[45] Nguyen T.T.X., Tomberlin J.K., Vanlaerhoven S. (2015). Ability of Black Soldier Fly (Diptera: Stratiomyidae) Larvae to Recycle Food Waste. Environ. Entomol..

[46] Myers H.M., Tomberlin J.K., Lambert B.D., Kattes D. (2008). Development of Black Soldier Fly (Diptera: Stratiomyidae) Larvae Fed Dairy Manure. Environ. Entomol..

[47] Banks I.J., Gibson W.T., Cameron M.M. (2014). Growth Rates of Black Soldier Fly Larvae Fed on Fresh Human Faeces and Their Implication for Improving Sanitation. Trop. Med. Int. Health.

[48] Silvennoinen K., Heikkilä L., Katajajuuri J.-M., Reinikainen A. (2015). Food Waste Volume and Origin: Case Studies in the Finnish Food Service Sector. Waste Manag..

[49] Kosobucki P., Chmarzynski A., Buszewski B. (2000). Sewage Sludge Composting. Pol. J. Environ. Stud..

[50] Hoffmann G., Schingnitz D., Bilitewski B. (2010). Comparing Different Methods of Analysing Sewage Sludge, Dewatered Sewage Sludge and Sewage Sludge Ash. Desalination.

[51] Erickson M.C., Islam M., Sheppard C., Liao J., Doyle M.P. (2004). Reduction of *Escherichia Coli* O157:H7 and *Salmonella Enterica* Serovar Enteritidis in Chicken Manure by Larvae of the Black Soldier Fly. J. Food Prot..

[52] Liu Q., Tomberlin J.K., Brady J.A., Sanford M.R., Yu Z. (2008). Black Soldier Fly (Diptera: Stratiomyidae) Larvae Reduce *Escherichia Coli* in Dairy Manure. Environ. Entomol..

[53] Belluco S., Losasso C., Maggioletti M., Alonzi C.C., Paoletti M.G., Ricci A. (2013). Edible Insects in a Food Safety and Nutritional Perspective: A Critical Review. Compr. Rev. Food Sci. Food Saf..

[54] Charlton A.J., Dickinson M., Wakefield M.E., Fitches E., Kenis M., Han R., Zhu F., Kone N., Grant M., Devic E. (2015). Exploring the Chemical Safety of Fly Larvae as a Source of Protein for Animal Feed. J. Insects Food Fee.

[55] Pas C., Brodeur D., Deschamps M.-H., Lebeuf Y., Adjalle K., Barnabé S., Eeckhout M., Vandenberg G., Vaneeckhaute C. (2022). Valorization of Pretreated Biogas Digestate with Black Soldier Fly (*Hermetia illucens*, L.; Diptera: Stratiomyidae) Larvae. J. Environ. Manag..

[56] Barragan-Fonseca K.B., Dicke M., van Loon J.J.A. (2018). Influence of Larval Density and Dietary Nutrient Concentration on Performance, Body Protein, and Fat Contents of Black Soldier Fly Larvae (*Hermetia illucens*). Entomol. Exp. Appl..

[57] Skřivan M., Englmaierová M., Skřivanová V. (2010). Effect of Different Phosphorus Levels on the Performance and Egg Quality of Laying Hens Fed Wheat- and Maize-Based Diets. Czech J. Anim. Sci..

[58] Nys Y. (1999). Nutritional Factors Affecting Eggshell Quality. Czech J. Anim. Sci..

[59] Crenshaw T.D. (2000). Calcium, Phosphorus, Vitamin D, and Vitamin K in Swine Nutrition. Swine Nutrition.

[60] Zelenka J., Heger J., Zeman L. (2007). Doporučený Obsah Živin v Krmných Směsích a Výživná Hodnota Krmiv pro Drůbež.

[61] Andrews J.W., Murai T., Campbell C. (1973). Effects of Dietary Calcium and Phosphorus on Growth, Food Conversion, Bone Ash and Hematocrit Levels of Catfish. J. Nutr..

[62] Rama Rao S.V., Ravindra Reddy V., Ramasubba Reddy V. (1999). Enhancement of Phytate Phosphorus Availability in the Diets of Commercial Broilers and Layers. Anim. Feed Sci. Technol..

[63] Lawlor P.G., Lynch P.B., Caffrey P.J., O’Reilly J.J., O’Connell M.K. (2005). Measurements of the Acid-Binding Capacity of Ingredients Used in Pig Diets. Ir. Vet. J..

[64] Johannsen O.A. (1922). Stratiomyiid Larvae and Puparia of the North Eastern States. J. N. Y. Entomol. Soc..

[65] Dow J.A. (2017). The Essential Roles of Metal Ions in Insect Homeostasis and Physiology. Curr. Opin. Insect Sci..

[66] Tschirner M., Simon A. (2015). Influence of Different Growing Substrates and Processing on the Nutrient Composition of Black Soldier Fly Larvae Destined for Animal Feed. J. Insects Food Feed.

[67] Bessa L.W., Pieterse E., Marais J., Dhanani K., Hoffman L.C. (2021). Food Safety of Consuming Black Soldier Fly (*Hermetia Illucens*) Larvae: Microbial, Heavy Metal and Cross-Reactive Allergen Risks. Foods.

[68] Elechi M.C., Kemabonta K.A., Ogbogu S.S., Orabueze I.C., Adetoro F.A., Adebayo H.A., Obe T.M. (2021). Heavy Metal Bioaccumulation in Prepupae of Black Soldier Fly *Hermetia illucens* (Diptera: Stratiomyidae) Cultured with Organic Wastes and Chicken Feed. Int. J. Trop. Insect Sci..

[69] Hu C., Yang L., Wang H., Xiao X., Wang Z., Gong X., Liu X., Li W. (2023). Analysis of Heavy Metals in the Conversion of Lake Sediment and Restaurant Waste by Black Soldier Fly (*Hermetia illucens*). Front. Bioeng. Biotechnol..

[70] Bohm K., Hatley G.A., Robinson B.H., Gutiérrez-Ginés M.J. (2022). Black Soldier Fly-Based Bioconversion of Biosolids Creates High-Value Products with Low Heavy Metal Concentrations. Resour. Conserv. Recycl..

[71] Wu N., Wang X., Xu X., Cai R., Xie S. (2020). Effects of Heavy Metals on the Bioaccumulation, Excretion and Gut Microbiome of Black Soldier Fly Larvae (*Hermetia illucens*). Ecotoxicol. Environ. Saf..

[72] Alaaeldin Abdelfattah E., Renault D. (2023). Does the Presence of Heavy Metal and Catechol Contaminants in Organic Waste Challenge the Physiological Performance of the Bioconverter *Hermetia illucens*?. J. Insect Physiol..

[73] Gao Q., Wang X., Wang W., Lei C., Zhu F. (2017). Influences of Chromium and Cadmium on the Development of Black Soldier Fly Larvae. Environ. Sci. Pollut. Res..

[74] Boykin K.L., Carter R.T., Butler-Perez K., Buck C.Q., Peters J.W., Rockwell K.E., Mitchell M.A. (2020). Digestibility of Black Soldier Fly Larvae (*Hermetia illucens*) Fed to Leopard Geckos (*Eublepharis macularius*). PLoS ONE.

[75] Čičková H., Newton G.L., Lacy R.C., Kozánek M. (2015). The Use of Fly Larvae for Organic Waste Treatment. Waste Manag..

